# Nanostructured Cellulose-Based Sorbent Materials for Water Decontamination from Organic Dyes

**DOI:** 10.3390/nano10081570

**Published:** 2020-08-10

**Authors:** Laura Riva, Nadia Pastori, Alice Panozzo, Manuela Antonelli, Carlo Punta

**Affiliations:** 1 Department of Chemistry, Materials, and Chemical Engineering “G. Natta” and INSTM Local Unit, Politecnico di Milano, Piazza Leonardo da Vinci 32, I-20133 Milano, Italy; laura2.riva@polimi.it (L.R.); nadia.pastori@polimi.it (N.P.); alice.panozzo@mail.polimi.it (A.P.); 2 Department of Civil and Environmental Engineering, Politecnico di Milano, Piazza Leonardo da Vinci 32, I-20133 Milano, Italy; 3 Centro Nazionale Ricerche (C. N. R.) Istituto di Chimica del Riconoscimento Molecolare (ICRM), 20131 Milan, Italy

**Keywords:** nanostructured materials, sorption, organic dyes, wastewater treatment, nanocellulose

## Abstract

Nanostructured materials have been recently proposed in the field of environmental remediation. The use of nanomaterials as building blocks for the design of nano-porous micro-dimensional systems is particularly promising since it can overcome the (eco-)toxicological risks associated with the use of nano-sized technologies. Following this approach, we report here the application of a nanostructured cellulose-based material as sorbent for effective removal of organic dyes from water. It consists of a micro- and nano-porous sponge-like system derived by thermal cross-linking among (2,2,6,6-Tetramethylpiperidin-1-yl)oxyl (TEMPO)-oxidized cellulose nanofibers (TOCNF), branched polyethylenimine 25 kDa (bPEI), and citric acid (CA). The sorbent efficiency was tested for four different organic dyes commonly used for fabric printing (Naphthol Blue Black, Orange II Sodium Salt, Brilliant Blue R, Cibacron Brilliant Yellow), by conducting both thermodynamic and kinetic studies. The material performance was compared with that of an activated carbon, commonly used for this application, in order to highlight the potentialities and limits of this biomass-based new material. The possibility of regeneration and reuse of the sorbent was also investigated.

## 1. Introduction

The use of engineered nanomaterials (ENMs) to clean-up polluted media, including groundwater and wastewater, has attracted more and more attention in the last decade [1]. This approach offers the possibility to take advantage of the high reactivity and high surface area of nanomaterials, opening the way towards more effective and economically sustainable remediation processes.

Nevertheless, the use of ENMs generates concerns associated with the potential risk for humans and environment, as the (eco-)toxicological impact of these solutions is often underestimated [2].

Recently, we proposed a systematic approach for possibly overcoming this issue, which consists of the use of sustainable and bio-based nanomaterials as building blocks for the design of nano-structured and nano-porous sorbents, capable of taking advantage of the intrinsic nano-dimension of the network, while overpassing the risk of ENM release and migration [3].

In accordance with this strategy, we first identified polysaccharides, and cellulose in particular, as ideal sources, often derived from discharged biomass, for the extraction of nano-sized particles to be further processed [4].

Cellulose represents the most abundant biodegradable and renewable polymer source in the biosphere, with an annual production estimated as over 7.5 × 10^10^ tons. This almost inexhaustible sustainable polymer possesses unique chemical, physical, and mechanical properties, which have suggested its use for the production of a wide range of materials [5].

Moreover, it is possible to cleave cellulose hierarchical structure in order to obtain nanocellulose (NC) in the form of nanocrystals (cellulose nanocrystals (CNC)) and nanofibers (cellulose nanofibers (CNF)). NC has been widely proposed as building block for the design of a wide range of sorbent materials to be used in wastewater treatment [6,7,8].

Among the several mechanical and chemical approaches for NC extraction, in recent years we focused on the one we considered the most effective and economically convenient, consisting of the selective oxidation of C6 alcoholic groups of the cellulose glucopyranose units to the corresponding carboxylic acids [9,10]. This transformation is mediated by 2,2,6,6-tetramethylpiperidine 1-oxyl free radical (TEMPO)/NaClO/NaBr system [9,10], and allows to promote nanodefibrillation at basic pH, thanks to the electrostatic repulsion among negatively charged TEMPO-oxidized CNF (TOCNF), due to the deprotonation of carboxylic moieties. The obtained nanofibers present a diameter in a range of 5–100 nm and a length of several microns.

Following the strategy previously described, in 2015 we reported a new class of nanostructured sorbent cellulose nanosponges (CNS), obtained by thermally promoting the cross-linking between TOCNF and branched polyethyleneimine 25 kDa (bPEI), a polymer bearing a high amount of primary, secondary, and tertiary amino groups, thanks to which it is able to interact with a wide range of ions and molecules [11].

In recent years, we modified the CNS formulation by adding an optimized amount of citric acid (CA) as an additional source of carboxylic groups, in order both to strength the mechanical properties of the final material by increasing cross-linking nodes [12], and to better fix bPEI into the network, so that it was also possible to reduce the amount of the same polymer and to support an eco-safe and sustainable design [13,14]. Moreover, we also demonstrated the versatility of the system, with the possibility of grafting bPEI with suitable moieties before undergoing cross-linking, in order to give the material additional functional properties, such as the sensing of fluoride ions [15,16].

Due to the chelating action of amino-groups, CNS have shown superb performances as heavy metals sorbents from both fresh- [11] and seawater [13,17]. On the contrary, the investigation into the behavior of this sorbent material with organic pollutants was limited to few examples, namely phenols, and simply based on an acid–base interaction.

However, by considering the morphology of the sponge, characterized by a high micro- and nano-porosity, as evidenced in previous works [12,18] and better discussed later on, we envisioned the possibility of exploiting the potential of CNS, and to better clarify the possible mechanisms of interaction between the sorbent and the selected organic pollutants.

Anionic organic dyes were chosen as model molecules, due to the continuous request for new and more sustainable technologies for treating a multiplicity of colored industrial wastewater, whose discharge in superficial water bodies can significantly impair freshwater use.

Nowadays, over 100,000 dyes are commercially available, with 7 × 10^5^ tons/year of dyestuff being produced [19]. Synthetic dyes are widely used in several industrial fields, including food, rubber, paper, cosmetic, pharmaceutical, automotive, and textile production chains. Inevitably, a certain fraction of dyes ends up in the washing water during the process and it has to be removed before release in the environment, due to the potential toxicity and negative effects on the aquatic ecosystem.

Due to their complex and variable chemical structure, many dyes are difficult to be removed by following a standard photo- and/or oxidative degradation approach, and sorption is often the process selected for wastewater treatment, with activated carbon mainly used for this purpose. In fact, sorption onto activated carbon is a well-established process, effective at removing a wide range of molecules, including color-responsible molecules from textile wastewater. Moreover, recently it has been reported that the efficiency of this process can be improved by driving the growth of nanoparticles into activated carbon pores, so that it is possible to combine sorption and degradation of the organic dye pollutant [20]. More generally, sorption represents a simply managed process, with no important drawbacks and a relative low cost, compared to other processes, such as ozonation.

In 2016, Zhu and coworkers first reported the grafting of cellulose with hyperbranched polyethyleneimine for the selective sorption of a wide range of anionic and cationic organic dyes [21]. The material was obtained by NaIO_4_-mediated oxidation to afford dialdehyde cellulose, which in turn was reacted with bPEI in ethanol. While the approach is quite interesting, the synthetic procedure required controlled and anhydrous conditions and the use of flammable solvents, which could limit the scalability of the synthesis. One year later, Wang and coworkers reported the use of TEMPO-oxidized cellulose membranes modified with linear PEI to remove both anionic (xylenol orange (XO)) and cationic (methylene blue (MB)) dyes from wastewater [22]. However, in this procedure the cross-linking is obtained by means of glutaraldehyde, which is known to be toxic. In both cases a good sorption performance was observed, and it was ascribed to an electrostatic interaction between the cationic polymer linked to cellulose and the dyes, with a sorption efficiency depending on the charge present on the surface. This aspect somehow limits the operating conditions of the process, as it would require wastewater pH adjustment before (and then, after) sorption treatment, with a consequent increase in the economic impact.

Herein, we report an investigation on the sorption efficiency of CNS towards four commercially available and highly used organic dyes (Naphthol Blue Black (NBB), Orange II Sodium Salt (OSS), Brilliant Blue R (BB), Cibacron Brilliant Yellow (CBY)). All the selected dyes presented a similar structure but differed in terms of molecular weight and the number of sulphate functional groups. The results in terms of sorption efficacy and regeneration efficiency were correlated with both the porosity of the sorbent sponge and the chemical and dimensional differences of the dyes, much more than on the charge present on the material. In fact, and quite surprisingly, CNS performed even better at a slightly basic pH, that is, one above the value of point of zero charge. Moreover, a comparison with an activated carbon could highlight the potentialities and limits of the proposed solution.

## 2. Materials and Methods

All the reagents were purchased from Sigma-Aldrich (Milano, Italy). Cotton linters was provided by Bartoli Spa paper mill (Capannori, Lucca, Italy). Deionized water was produced with a Millipore Elix^®^ Deionizer with Progard^®^ S2 ion exchange resins. UV spectra and data were recorded on a V-600 Series UV-vis spectrophotometer from JASCO (Cremella (LC), Italy). Other equipment used in the procedures include a Branson Sonifier 250 equipped with a 6.5 mm probe tip, a Heldolph multi-reax shaker (Schwabach, Germany) and a SP Scientific BenchTop Pro Lyophilizer (Perugia, Italy).

### 2.1. TOCNF Synthesis and Titration

Cellulose was oxidized to a degree of 1.546 mmol_COOH_/g_TOCNF_ according to a procedure previously reported in the literature [9,10]. Briefly, cotton linters (190 g) were dispersed in deionized water produced with a Millipore Elix^®^ Deionizer with Progard^®^ S2 ion exchange resins (Milan, Italy) (total volume 5.7 L), in the presence of TEMPO (2.15 g, 13.8 mmol) and KBr (15.42 g, 129 mmol) and a solution of NaClO (12.5% *w*/*w* aqueous solution, 437 mL) was slowly added under vigorous stirring. During oxidation, pH was maintained in the range of 10.5-11 by dripping 4 N NaOH water solution. The solution was maintained stirred for 16 h. Oxidized cellulose nanofibers were aggregated by using concentrated HCl and then washed with deionized water on a Büchner funnel to reach a neutral pH (see Supplementary Materials for further information).

To estimate the concentration of carboxyl groups on the cellulose structure after oxidation, a titration was performed with 0.1 N NaOH water solution, using phenolphthalein as a colorimetric indicator. The first step was the titration of NaOH 0.1 N. The detailed procedure and the equation used to calculate the concentration of the carboxyl groups are reported in Supplementary Materials.

### 2.2. Synthesis of CNS

CNS were synthesized according to the procedure previously reported [13]. First, 3.5 g of TOCNF were suspended in deionized water, adding a stoichiometric amount of granular NaOH. The suspension was ultrasonicated with a Branson Sonifier 250 equipped with a 6.5 mm probe tip to further promote the separation of the nanofibers, obtaining a homogeneous solution, which was then acidified with 12 M HCl, filtered on a Büchner funnel under vacuum, and washed with deionized water until neutrality. Then, two aqueous solutions of 25 kDa bPEI (3.5 g of bPEI in 10 mL) and anhydrous citric acid (CA) (0.896 g in 10 mL) were slowly added to the TOCNF solution, while continuously stirring until obtaining a white and homogeneous hydrogel, which was placed in well-plates, quickly frozen at −35 °C, freeze-dried for 48 h using a SP Scientific BenchTop Pro Lyophilizer (at –52 °C temperature and 140 μbar pressure) and then thermally treated in the laboratory oven at a maximum temperature of 102 °C for 16 h. At the end of the process, CNS was grinded with a mortar and then washed with water to remove the excess bPEI (for further information, see Supplementary Materials). The particle size distribution of the grinded CNS powder was measured in the Laboratory Chemical Analysis (LAC) of Politecnico di Milano by means of a Malvern Mastersizer 3000 Particle Size Analyzer (Malvern, UK) with Fraunhofer modeling, which considers opaque non-spherical particles. The CNS powder was suspended under stirring in 500 mL of water to reach an obscuration level in the range of 8–12%.

### 2.3. Point of Zero Charge (PZC) Calculation

The pH of the point of zero charge (pH_PZC_), namely the pH above which the total surface of the sorbent material is negatively charged, was measured by the pH drift method [23]. For this purpose, 20 mL of a 0.01 M NaCl solution was placed in a Falcon vial and N_2_ was bubbled through the solution to steady the pH by preventing the dissolution of CO_2_ in the solution. The pH was then adjusted to selected initial values between 2 and 12, by dripping HCl 0.01 N or NaOH 0.01 N, and the sorbent (0.06 g) was added to the solution. The final pH, reached after 4 h, was measured and plotted against the initial pH. The pH at which the curve crosses the bisector pH(final) = pH(initial) is the pH_PZC_ of the given sorbent. The considered sorbents were CNS and the activated carbon SAE SUPER, provided by Norit (Italy) (see Supplementary Materials for general characteristics).

### 2.4. Preliminary Sorption Tests

**Figure 1:**
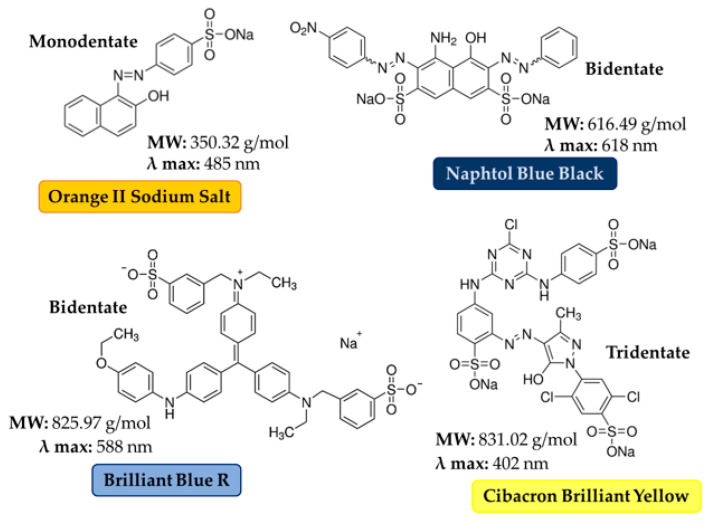
Chemical structure and properties of the dyes used for the sorption tests.

The tests were carried out by dipping 12 mg of CNS powder in 15 mL of aqueous solutions of the selected dye for 24 h, under static conditions and at room temperature in a Falcon vial. The selected dyes are reported in Figure 1. The concentration of the buffer dye solution was selected considering the extinction coefficient of each dye (20 mg/L for OSS and NBB, 100 mg/L for BB and CBY) and the type of buffer was chosen according to the solubility of the dye in the buffer solution at room temperature (OSS: 116 g/L, NBB: 30 g/L, BB: 70 g/L, CBY: 50 g/L). Tests at pH 5.5 were carried out in piperazine and citrate buffer. Tests at pH 7.6 were conducted in deionized water using the normal buffering power of CNS. Each sample has been reproduced in triplicate. After 24 h, one collection was taken from each sample and analyzed by UV analysis. UV-Vis spectra (Figure S1), characteristic λ_max_ (Table S1), extinction coefficients (Table S2) and calibration lines (Figures S2–S5) for all the four dyes are reported in Supplementary Materials.

### 2.5. Isotherms and Kinetics

Isotherm and kinetic sorption tests were carried out under dynamic conditions (using the shaker at 450 rpm) at room temperature (25 °C). Isotherm tests were carried out by maintaining a constant sorbent quantity and solution volume throughout the data gathering (24 h), while changing the solution concentration. Eight different concentrations were tested for each dye and three trials were carried out for each concentration. The sponge-to-solution ratio used was 12 mg of CNS/15 mL of mono-contaminated dye solution (0.8 mg/mL). The selected concentrations for each dye are described in Table S3 in Supplementary Materials. The absorbance of the solutions was measured at time zero and after 24 h.

As for kinetic tests, the initial concentrations for each dye are reported in Table 1. The trials were performed under dynamic conditions and at room temperature, as described above. Samples were shaken and analyzed after 15, 30, 45, 60 and 90 min, and 2, 3, 4, 6, 8 and 24 h. A volume of 25 mL of solution was used for each test, to allow withdrawals from the solution 11 times, keeping the total diminution of the volume below 10%. The quantities of sorbent for each kinetic test were 20 mg for the lowest concentration and 40 mg for the highest concentration.

### 2.6. Desorption and Reusability Tests

Tests were conducted to evaluate the possibility of reusing the sponges. At first, colored CNS was produced by leaving white CNS powder in contact with a solution of the selected dye in static conditions (5 g/L concentration, 30 mL, 200 mg of CNS) for 24 h, then filtering it on a Büchner funnel and washing it with deionized water.

The desorption test was conducted with HCl 0.1 N and NaOH 0.1 N. A total of 20 mg of colored CNS was soaked in 20 mL of each solution under static conditions at room temperature for 24 h. After the first test, which decreed the efficiency for only the NaOH solution, three different molar concentrations of NaOH were compared—0.5, 0.1 and 0.05 N—following the same discoloration procedure. Other types of alkaline solutions were tested using the previously reported conditions: the test was repeated with triethylamine (TEA), NH_3_ 30% aqueous solution and KOH 0.1 N aqueous solution.

The reusability test consisted of a sorption test conducted on the decolored sponge. The test was carried out only on the OSS discolored CNS and following the same procedure as for previous sorption tests: 12 mg of sorbent in 15 mL of 20 mg/L solution of OSS dye for 24 h under static conditions at room temperature. Five sorption–desorption cycles were carried out. For this purpose, 100 mg of OSS colored CNS were soaked in 110 mL of NaOH 0.05 N. The resulting sponge was then vacuum-filtered with the aid of a Büchner funnel and washed with deionized water until neutrality. Once dried, the sponge was weighted for the next phase of sorption with a constant sponge-to-solution ratio of 0.8 mg/mL. Each sample was carried out in triplicate. After 24 h of static sorption at room temperature, absorbance was analyzed. The sponge was then gravity-filtered and air-dried before the new desorption phase, carried out with NaOH 0.05 M solution.

### 2.7. Comparison between CNS and Activated Carbons

A comparison test was carried out by evaluating the sorption capacity of the activated carbon SAE SUPER. The experimental setup was the same as the previous tests, using a sorbent-to-solution ratio of 0.8 mg/mL. Two initial concentrations were tested for each dye (C_0_ low and C_0_ high, reported in Table 1). This test was carried out in dynamic conditions and equilibrium was reached after 24 h in the multi reax shaker at 450 rpm. Filtration via syringe filter was required for this test due to the fine particulate dispersion of activated carbons in the solution.

Kinetic tests were carried out using SAE SUPER activated carbon in OSS solutions at high and low concentrations. For the high-concentration trial, 240 mg of SAE SUPER activated carbon were dispersed in 150 mL of 800 mg/L OSS solution. Three trials were prepared and agitated by magnetic stirring. A volume of 1 mL was withdrawn for each measurement, filtered through a cotton filter and opportunely diluted. Withdrawals were performed after 15, 30, 45, 60 and 90 min, 2, 3 and 24 h. The total amount of withdrawn solution after eight samplings was still lower than 10% of the total volume of the solution.

## 3. Results and Discussion

### 3.1. CNS Synthesis and Characterization

**Scheme 1:**
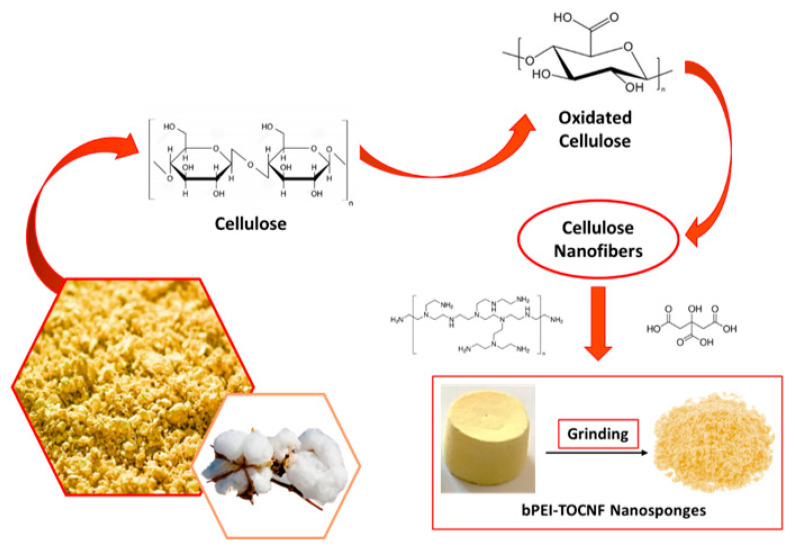
Preparation of CNS starting from cellulose-based sources.

CNS were synthesized according to Scheme 1, following a two-step protocol. TOCNF and bPEI were first mixed in deionized water in a 1:1 weight ratio, and CA (18% with respect to primary amino groups of bPEI) was added, with the final aim to better trap bPEI in the final network by increasing the concentration of carboxylic groups. The same result cannot be achieved by raising the content of carboxylic units on the nanofiber, as more severe oxidation conditions lead to depolymerization rather than further selective conversion of C6 alcoholic groups. In a second thermal step, the resulting hydrogel was transferred in well-plates, used as molds, lyophilized and then heated in an oven at about 100 °C, in order to favor the cross-linking between the carboxylic groups of TOCNF and the primary amines of bPEI. Finally, CNS was grinded in a mortar before use, in order to increase sorption performance.

A complete chemical characterization of the resulting sponges was reported in a previous paper [12]. The formation of amide bonds after thermal treatment was evidenced by FT-IR analysis, with an increase in the peak at 1664 cm^−1^ (−C=O stretching of the amide bonds), directly related to an increase in CA content in the formulation, as also confirmed by ^13^C CP-MAS solid-state NMR. The role of CA in better fixing bPEI in the network was also quantitatively confirmed by processing data derived from the elemental analysis of different nanosponges, prepared by varying the content of this tri-carboxylic molecule in starting solution. This optimization study, also supported by an eco-toxicology evaluation of the materials [13], led to the CNS formulation herein investigated.

**Figure 2:**
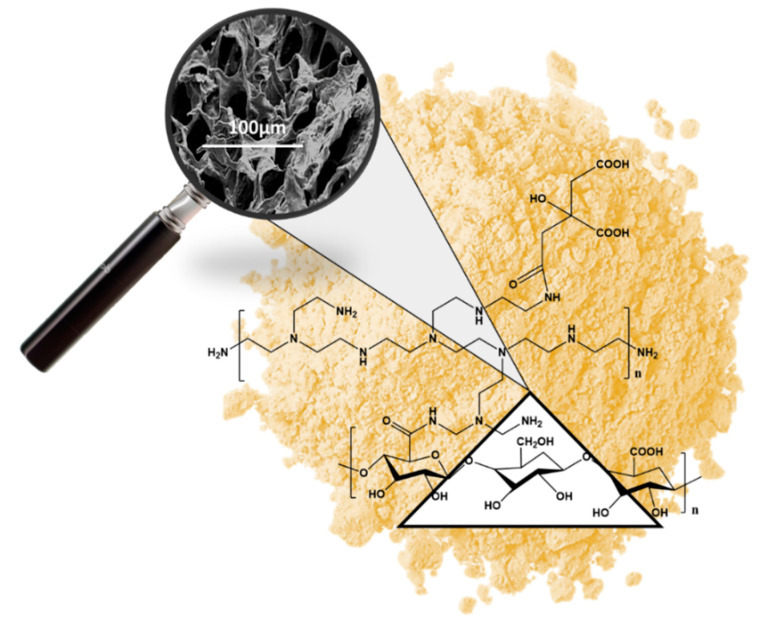
Inner structure of grinded CNS analyzed by Scanning Electron Microscopy.

CNS exhibit a high micro-porosity with pore sizes in the range of 10–100 μm, as observed by scanning electron microscopy (SEM) (Figure 2). Pores are characterized by a two-dimensional sheetlike morphology, often reported in the literature for cellulose-based aerogels prepared by freeze-drying aqueous suspensions. According to this approach, ice crystals act as templates for pores’ generation, preventing the formation of occlusions and guaranteeing complete penetrability of the structure [24].

Microcomputed tomography (μ-CT) analysis previously reported [12] also indicated that CNS has a porosity of 70–75%, and a trabecular inner structure with an average trabecular thickness of about 30–40 μm and a trabecular separation of about 70–75 μm.

Moreover, we recently provided experimental evidence of nano-porosity in the network, by means of small angle neutron scattering (SANS) analysis of water nanoconfinement geometries in the sorbent material [18]. The analysis of the experimental data allowed us to measure the short-range correlation length, which resulted in a range between 25 and 35 Å. In addition, a more recent combined investigation of the FTIR-ATR spectra of CNS hydrated with H_2_O and D_2_O allowed to detect a supercooled behavior of entrapped water molecules, supporting the idea of a nano-confinement for water in these systems.

The high porosity and the wide pores’ dimensional dispersion would suggest a high diffusivity of solutes in the material, which could be however affected by their structure and dimension.

### 3.2. Sorption Tests

#### 3.2.1. First Sorption Screening for All Dyes

**Figure 3:**
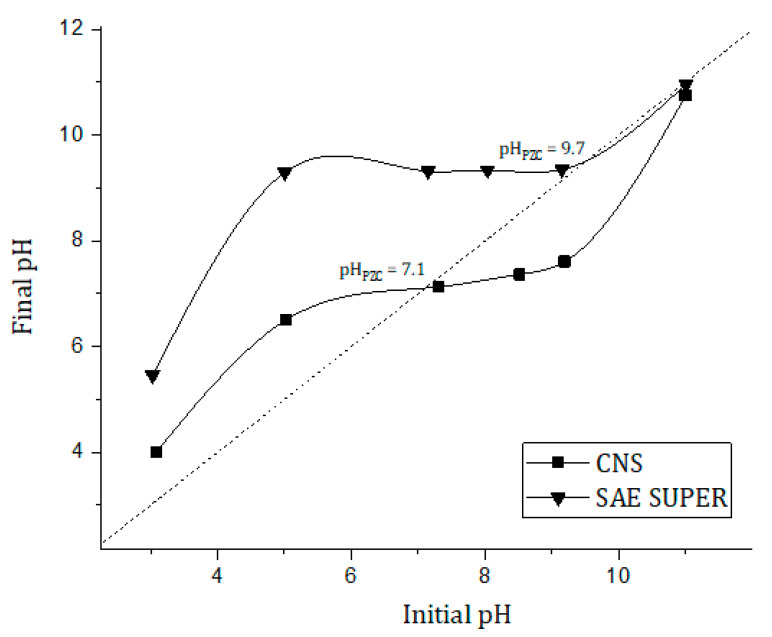
pH drift method plot to determine the pH_PZC_ for CNS and SAE SUPER-activated carbon.

Before starting sorption experiments, we determined the pH_PZC_ for CNS, which was pH 7.1 (Figure 3). This value indicates that at alkaline pH, the material is negatively charged.

According to this result, we selected two different pH ranges for performing preliminary sorption tests. At pH 7.5−7.8, CNS should be negatively charged. This condition limits the electrostatic interaction with the negatively charged dyes and could be considered as not ideal for our purpose. However, it falls into the typical pH ranges of textile wastewater. Moreover, this is the buffer directly generated by CNS powder once dispersed in deionized water. On the contrary, at pH 5.5, CNS should be positively charged. However, at this pH value, both OSS and NBB showed a poor solubility, which limited the interest in this experimental set.

**Figure 4:**
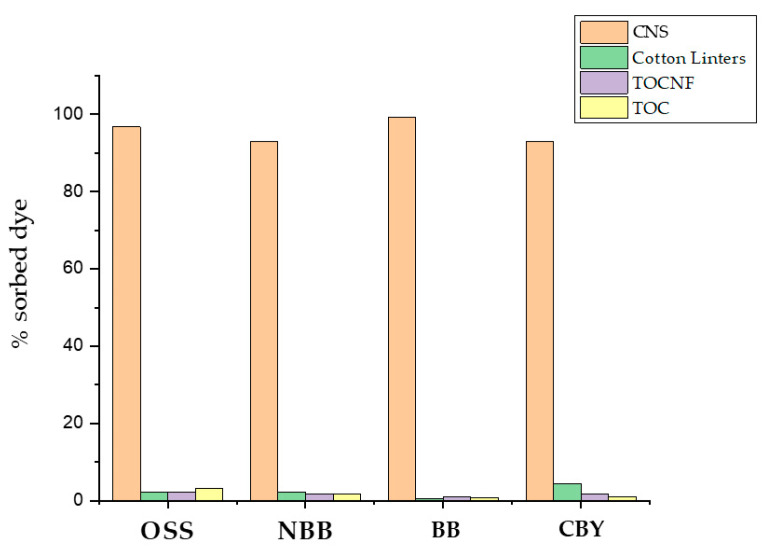
Comparison between sorption capacities of CNS and different cellulose forms (cotton linters, TEMPO-oxidised cellulose (TOC), TOC nanofibers (TOCNF)). Data are reported as the percentage of dye adsorbed at the end of the test with respect to the total amount of dye in solution at t_0_.

Preliminary results at pH 7.6 confirmed a good sorption performance of CNS towards all the dyes under investigation (Table 2). We operated at two different dyes’ concentrations (C_0_) for NBB and OSS (20 mg/L) and BB and CBY (100 mg/L), due to the different extinction coefficients of each dye. Obviously, sorption capacity (q_e_) value, which is the amount of contaminant taken up by the sorbent per unit mass of the sorbent, was dependent on C_0_, so that a comparison among dyes is not correct at this stage. As expected, cellulose alone, regardless of its original form (cotton linters, TEMPO-oxidized cellulose (TOC), or TOCNF) did not perform any sorption (Figure 4), confirming the crucial role of bPEI in CNS network. Moreover, sorption tests at pH 5.5 conducted on BB and CBY provided a q_e_ value quite similar to that measured at pH 7.6 (96.31 and 110.53 mg/g, respectively), indicating that CNS charge is not crucial for the sorption performance.

#### 3.2.2. Isotherm Models

**Figure 5:**
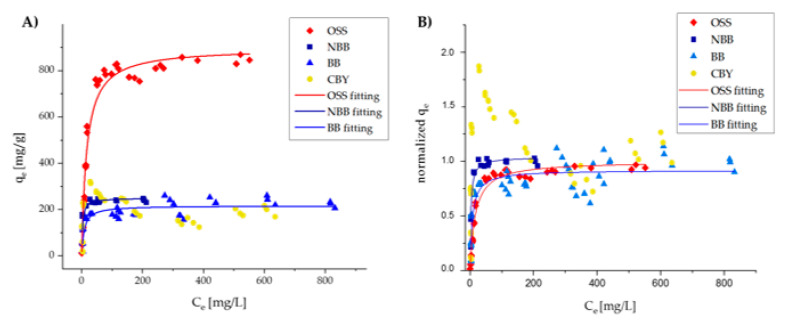
Graph showing the data collected for the calculation of isotherm curves. The x-axis shows the concentration at equilibrium (C_e_) expressed in mg/L, while the y-axis shows the capacity at equilibrium (q_e_) expressed in mg/g and not normalized (**A**) and normalized for the maximum capacity (Q_max_) (**B**).

Isotherm experiments were conducted maintaining constant the amount of CNS in solution, and progressively increasing dye concentration [25]. Data were modeled via non-linearized methods [26], originally considering three different models (Langmuir, Freundlich, and Dubinin–Radushkevic) [27], and then selecting the Langmuir one, which better fitted collected data. This model (Equation (1)), which assumes that the sorbent is coated by a monolayer of the adsorbate, correlates the sorption capacity of the sorbent material (q_e_)—calculated as mg of pollutants sorbed per g of sorbent material—with the concentration of the pollutant in the solution at the equilibrium (C_e_). All data collected and Langmuir fittings for OSS, NBB and BB are reported in Table 3 and Figure 5.

(1) $$q_{e}=\frac{Q_{\max}{\mathrm{KC}}_{e}}{1+{\mathrm{KC}}_{e}}$$

The graph’s and the main fitting’s statistics for Freundlich and Dubinin–Radushkevic models are reported in the Supplementary Materials (Tables S3–S5 and Figures S6–S8). Langmuir isotherm is a model for monolayer-localized sorption on a homogeneous surface containing a finite number of identical sites; probably for this reason, it is the one providing the better description of experimental data of dye removal by nanosponge, since neither Freundlich nor Dubinin–Radushkevic models assume a homogeneous surface or constant sorption potential. In fact, the Freundlich’s empirical formula accounts for the sorption on heterogeneous surfaces as well as multilayer sorption on microporous structure, which is not the case of CNS, which is characterized by a homogenous nanoporous structure. Similarly, the Dubinin–Radushkevic model is more general than the Langmuir one, since it does not assume a homogeneous surface or constant adsorption potential, meaning that it assumes a Gaussian energy distribution onto a heterogeneous surface. Consequently, these assumptions do not fully describe CNS characteristics. As for the parameters of the Langmuir model, Q_max_ represents the maximum sorption capacity of the sorbent, while K is related to the free energy of sorption, directly related to the affinity of the compound for the solid phase.

The OSS dataset was fitted with a very high accuracy. The very steep slope of the first portion of the curve indicates that the sorption efficiency (q_e_) would also be high at very low initial concentrations of pollutant, highlighting the high sorbent/solute affinity. Moreover, the maximum sorption capacity for this dye (Q_max_) was estimated to be very high compared to other similar sorbents [28].

The NBB dataset was not highly coherent with the Langmuir model. When operating at a C_0_ lower than 150 mg/L, the equilibrium concentration (C_e_) was constant and very close to 0. This behavior is typical of the chemisorption processes, which can occur in the presence of sulfonated dyes. By excluding the data that deviate from the Langmuir model, a quite accurate modeling can be obtained, where the slope in the first section is still very steep (suggesting excellent sorption capacity also at low initial concentrations). Q_max_ was significantly lower than that measured for OSS, but still in line with the results obtained for other similar sorbents [28].

For the BB dataset, the modelling was not deemed reliable, because of a quite low accuracy. However, general considerations can still be made. It can be observed that, very similarly to NBB, the slope of the initial section of the curve is very steep and the maximum sorption capacity is in line with the results reported in the literature [28].

A particular case is represented by CBY, for which an anomalous isotherm data trend makes the Langmuir model’s application impossible. As the equilibrium concentration grows, a very steep increase in the sorption capacity can be observed up to a maximum value, after which the sorption capacity decreases to a lower plateau value. This anomalous behavior can be attributed to the formation of solute–solute interactions stronger than the sorbent-solute ones at higher concentrations. The maximum sorption capacity value is of approximately 310 mg/g, but the sorption capacity at the plateau is of approximately 200 mg/g. Despite this irregular behavior, some similarities with the other dyes can be evidenced. In fact, in this case, the slope of the initial section of the curve is steep and the sorption capacity plateau value is comparable with those obtained for BB and NBB.

All these results suggest some preliminary considerations. A clear difference in Q_max_ values between OSS and the set of the other three dyes is observed. We identify two parameters which make these dyes different, namely the number of sulfonate units present into the molecular structure and the molecule dimension. The first parameter should affect the sorbent–solute interaction strength, as evidenced by the very steep slope at low C_0,_ especially for NBB, BB, and CBY, and the higher K values calculated for BB and NBB with respect to OSS (Table 4), with the latter bearing only one sulfonate group. Nevertheless, we assume also that the molecular size can play a key role in determining the diffusivity of the solute in the sponge. In fact, OSS (MW = 350.32 g/mol) is much smaller with respect to the other three dyes (NBB (MW = 616.49 g/mol), BB (MW = 825.97 g/mol) and CBY (MW = 831.02 g/mol)), and this probably favors its penetration in the sponge.

**Figure 6:**
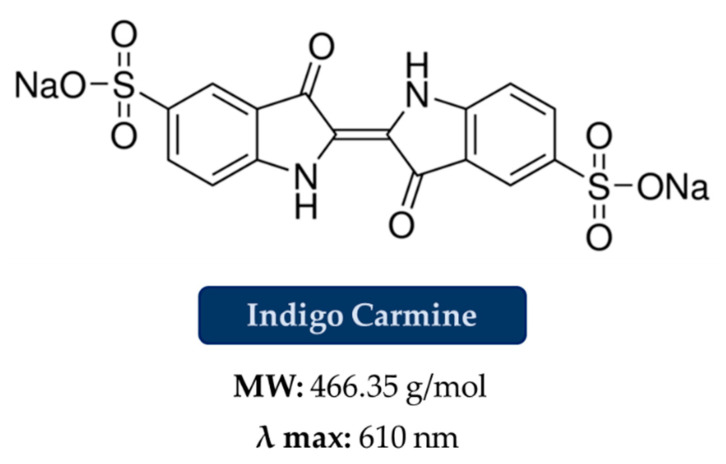
Chemical and sorption properties of Indigo Carmine.

To confirm our hypothesis, we carried out some sorption tests on solutions contaminated by Indigo Carmine (IC), [29] an organic dye with a structure quite similar to those previously analyzed (Figure 6), and which presents two sulfonate groups, but an MW of 466.35 g/mol, that is an intermediate value between OSS and the other dyes. A Q_max_ of 540 mg/g was obtained, which, as for the molecular weight, is an intermediate value between the Q_max_ of OSS and the Q_max_ of NBB, BB and CBY.

#### 3.2.3. Sorption Kinetics

Kinetic study is of great importance for the use of granular sorbent in the water treatment field, since the solute removal rate affects the reactor residence time required for completing sorption reactions and therefore for achieving the selected quality standard for treated water [30,31].

Kinetic experiments were conducted at two different C_0_ (see Table 1). The two initial concentrations were selected as the minimum UV detectable concentration (C_0_ low) and the minimum C_0,_ which can reach the Q_max_ value (C_0_ high) according to the Langmuir model for OSS, NBB and BB. As regards CBY, concentration and CNS amounts were selected in order to be in the same range as BB, due to its similar chemical structure.

**Figure 7:**
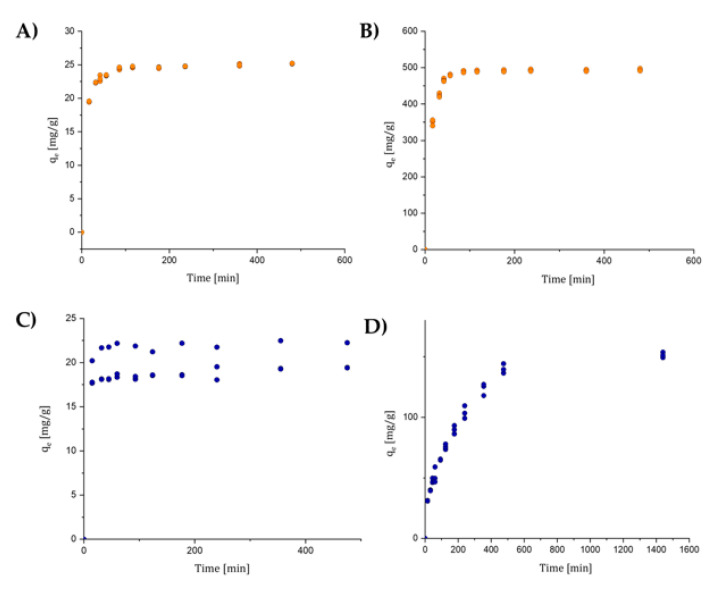
(**A**) OSS kinetic behavior. Initial concentration of 20 mg/L. (**B**) OSS kinetic behavior. Initial concentration of 800 mg/L. (**C**) NBB kinetic behavior. Initial concentration of 20 mg/L. (**D**) NBB kinetic behavior. Initial concentration of 250 mg/L.

**Figure 8:**
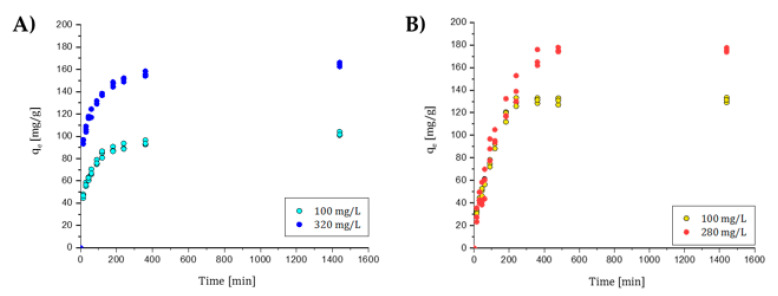
(**A**) BB kinetic behavior. Initial concentration of 100 and 320 mg/L. (**B**) CBY kinetic behavior. Initial concentration of 100 and 280 mg/L.

All the graphs related to the sorption kinetic behavior for each dye are reported in Figure 7 and Figure 8.

OSS was tested at C_0_ low and C_0_ high (20 and 800 mg/L). At both concentrations, more than 80% and 70%, respectively, of the total dye sorbed was reached in the first 15 min, while 90% was reached after 1 h. This dye was the one with the best performance for both initial conditions. NBB was also tested at C_0_ low and C_0_ high (20 and 250 mg/L). At C_0_ low, more than 90% of the total dye sorbed was reached in the first 15 min. At C_0_ high, 50% was adsorbed after 2 h, while it required 8 h to reach 90% sorption efficiency.

For BB C_0_ low was 100 mg/L, while C_0_ high was 320 mg/L. In the first case, 50% of the maximum efficiency was reached in the first 15 min, while in the second case, 70% was reached in the same time. To reach 90%, 4 h were required in the first case, while in the second case only 3 h were sufficient. C_0_ low and C_0_ high for CBY were 100 and 280 mg/L. At the lowest concentration, 50% was reached after less than 90 min, while to reach 90%, 3 h were required. In the other case, 90 min were required to reach 50%, while 6 h were necessary to reach 90%. This dye was the one with the worst kinetic behavior. However, in all cases the sorption kinetic was quite fast, and almost immediate for low concentrations, which are, however, much more similar to those expected in wastewater.

All kinetics were analyzed to determine whether the data reflected a *pseudo first-order* model or a *pseudo second-order* model. The model that best fits all the kinetics datasets was the *pseudo second-order* one (Equation (2)), as was expected considering the results from isotherm experiments (see Table S7 in Supplementary Materials for the comparison between the two models). In fact, the *pseudo second-order* kinetic usually applies to chemisorption processes, in which solutes can react with more than one active site [31,32]. This is associated with two main assumptions: (i) the kinetic rate limiting step is a chemical reaction involving valent forces through sharing or the exchange of electrons, (ii) the sorption follows the Langmuir equation. CBY, which does not follow the Langmuir fitting regarding the isotherm fit, cannot be modelled either with first-order kinetics or second-order kinetics. Figures S9 and S10 in Supplementary Materials report the *pseudo second-order fitting* for OSS sorption at 20 mg/L and 800 mg/L initial concentrations, respectively.

Table 4 shows schematically the values obtained using the *pseudo second-order* model on all the dyes considered. In Section S5 of Supplementary Materials, the procedure and *pseudo second-order* fitting graphs are also shown, taking as an example the kinetics of OSS at 20 and 800 mg/L.

(2) $$\frac{dq_{t}}{dt}=k_{2}{\left(q_{eq}-q_{t}\right)}^{2}$$

### 3.3. CNS Regeneration

To better exploit the potentialities of CNS as a sorbent towards anionic dyes, we wanted to evaluate the possibility to regenerate and re-use the material after first sorption experiments.

#### 3.3.1. Desorption Tests

Alkaline washing of CNS sponges with NaOH water solution led to discoloration of the material, while desorption trials with acidic aqueous solutions provided poor results in terms of CNS regeneration. We hypothesized that the selected dyes, due to the presence of sulfonate groups, are much more soluble at alkaline pH and therefore, under these conditions, the solute–solvent interactions prevail on solute–sorbent ones. While dyes’ sorption can occur also at a slight basic pH, which is higher than the pH_PZC_ of CNS, thanks to Van der Waals interactions between the amino-groups of the sponge and the sulfonate moieties of the dye; once they are sorbed, an acidic treatment seems to enforce the solute–sorbent interaction, rather than promoting sponge regeneration.

Interestingly, while OSS-CNS were completely discolored through alkaline washing, NBB-, BB- and CBY-CNS showed only partial discoloration under the same treatment conditions. In this case, the different behaviors should be ascribed to the different number of sulfonate groups present on the organic dye molecules, rather than to the molecular dimensions. Polydentate dyes NBB and BB (bidentate) and CBY (tridentate) seem to have a stronger interaction with the sorbent if compared with OSS, which bears just one sulfonate group. Once again, this aspect can be explained by considering the K values reported in Table 4. As previously stated, this parameter is tightly related to the free energy of sorption, and corresponds to the affinity of the compound for the solid phase. By considering these values, we can notice that the K parameter for OSS is notably lower than those for NBB and BB, suggesting a stronger sorbent–molecule interaction for the latter, and so implying more difficulty in the desorption process, due to the higher energy required.

Once again, a similar desorption test conducted on CNS loaded with IC, a bidentate molecule with an MW lower than those of the other polydentate dyes, provided an incomplete regeneration, comparable with that of NBB, BB and CBY.

For OSS-CNS, the 0.05 N NaOH solution was sufficient to completely regenerate the material.

#### 3.3.2. Reusability Tests

Tests were performed on the OSS-CNS, due to its complete regeneration by alkaline treatment. This test consisted of several sorption–desorption cycles to evaluate the impact of repeated decoloring on the sorption efficiency of the sponge. Reusability efficiency was evaluated through five cycles and the results reported in Table 5 clearly show how the sorption capacity of the sponge is maintained at a constant after several regeneration cycles.

### 3.4. Comparison with Activated Carbons

A comparison test between sponges and an activated carbon was carried out. For the selected activated carbon, the PZC was calculated according to the procedure described in Section 2.3. Results are reported in Figure 3.

Contrary to the CNS, SAE SUPER is positively charged at the operating pH (7.5–7.8), thus favoring the interaction between the positive charge of the sorbent and the negative charge of the deprotonated SO_3_^−^ groups of the dye. A detailed set up for the comparison test is reported in Section 2.7. The sorption comparative results for low and high concentrations of the dyes are reported in Table 6.

It can be observed that the sorption capacity of the activated carbon at low concentrations is comparable to the one for CNS for all dyes. Regarding the high-concentration sorption tests, the sponge showed comparable performances to the activated carbon for NBB, while the sorption capacity towards OSS was double for the sponge compared to the activated carbon. CBY was sorbed better by the sponge than the activated carbon. A different behavior was displayed by BB, which showed clearly better performances for the activated carbon than for the nanosponge.

**Figure 9:**
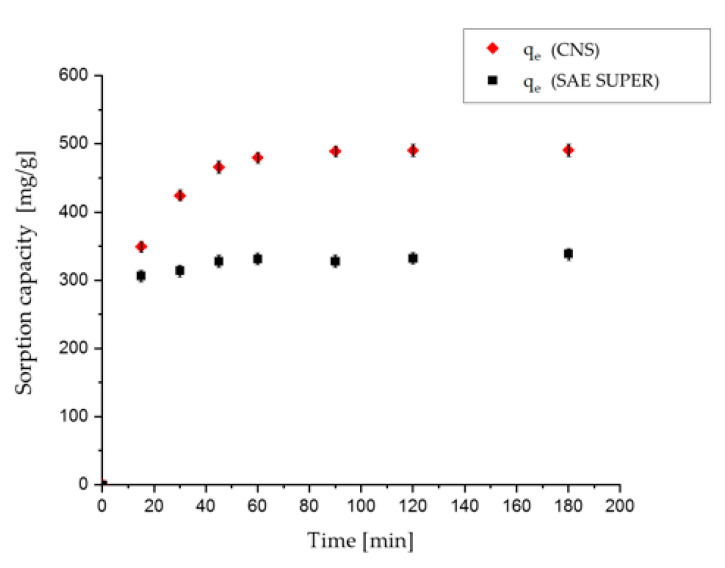
Comparison between SAE-activated carbon and sponge sorption kinetics for OSS dye at 800 mg/L concentration.

Kinetic studies were thus performed on SAE SUPER activated carbon for the sorption of 20 and 800 mg/L solutions of OSS dye. For the 800 mg/L trial with activated carbon, a plateau was reached in the first 30 min, while for the nanosponge one hour was required to reach the plateau. However, the plateau values obtained for the nanosponge were much higher than for the activated carbon. As regards the 20 mg/L trial, the first measurement for the SAE SUPER after 7 min was registered to be already below the UV detection limit. On the contrary, at this concentration, the kinetic behavior of the nanosponge took approximately 90 min to reach the same result. Figure 9 shows graphically the kinetic behavior of SAE SUPER and CNS in contact with an 800 mg/L solution of OSS.

The faster kinetics observed for the activated carbon with respect to the nanosponge could be due to different intra-particle diffusion resistances, considering the different porous structures of these two sorbents. In fact, in batch experiments, usually performed in turbulent conditions, the limiting step is usually diffusion into pores [33].

## 4. Conclusions

In this work, we reported the use of a nanostructured-cellulose-based sorbent material for water decontamination from anionic organic dyes. The material was prepared starting from biomass sources, by combining TEMPO-oxidized cellulose nanofibers, branched polyethyleneimine, and citric acid, and following a simple thermal protocol. The sorption performance was tested on four commercial dyes (OSS, BB, NBB, and CBY), differing for both molecular dimension and the number of sulfonate groups present onto the molecular structure (one for OSS, two for BB and NBB, three for CBY). The sorbent was effective also at a slightly basic pH, even if under these conditions the nanostructured sponge is negatively charged. This result suggested that the sorbent–solute interaction should not be simply ascribed to electrostatic attraction between opposite charges, but other intermolecular interactions could occur between the sulfonate groups of the dyes and the amino groups present on the nano-sponge. The role of bPEI in the network was crucial, as cellulose alone was not able to reproduce significant sorption.

Isotherm and kinetic investigation revealed a molecular-size dependence of sorption performance, as the smallest OSS is much more trapped on the material, probably because of the possibility of it being more diffused in the nano-porous network. Nevertheless, these studies also showed that the strength of sorbent–solute binding was higher when two or more sulfonate groups were present on the dye. This evidence was also confirmed by conducting regeneration and reusability tests, as once again OSS was much more easily removed from the nano-sponge under alkaline conditions, so that the sorbent system could be reused several times, by maintaining its sorption efficiency.

The dye-removal efficiency of the material herein described was compared to that of the commercially available activated carbon SAE SUPER. While with the nano-sponge the sorption kinetic was slightly slower, probably due to the nano-porous structure with respect to a microporous structure of the activated carbon, the sorption capacity was higher for all dyes except BB. In any case, the advantages of the use of this cellulose-based material can be found in its easy handling, reusability, eco-safety, and sustainability, as it can be produced from wasted biomass, following the virtuous route of the circular economy.

## Figures and Tables

**Table 1:** Values of initial concentrations (C_0_) and sponge quantities (m) for kinetic tests, where the solution volume was 25 mL. The same C_0_ values are also used for comparison tests between cellulose nanosponges (CNS) and activated carbon (Section 2.7).

Dye	C_0_ low		C_0_ high	
	C_0_ (mg/L)	m (mg)	C_0_ (mg/L)	m (mg)
OSS	20 ± 0.5	20 ± 0.1	800 ± 5.0	40 ± 0.1
NBB	20 ± 0.5	20 ± 0.1	250 ± 2.0 ^a^	40 ± 0.1
BB	100 ± 1.0	20 ± 0.1	320 ± 2.0	40 ± 0.1
CBY	100 ± 1.0	20 ± 0.1	320 ± 2.0	40 ± 0.1

^a^ The value of C_0_ high for comparison tests between CNS and activated carbon is 400 mg/L.

**Table 2:** Preliminary sorption tests at pH 7.6 starting from solutions at different dyes’ concentrations (C_0_).

Dye	q_e_ (mg/g)	C_0_ (mg/L)
OSS	22.58 ± 1.19	20 ± 0.5
NBB	23.85 ± 2.63	20 ± 0.5
BB	88.96 ± 4.23	100 ± 1.0
CBY	121.53 ± 4.59	100 ± 1.0

**Table 3:** Estimation of Q_max_ and K parameters according to the Langmuir isotherm model for OSS, NBB and BB. The number of experimental data used for each dye is reported in column N.

Dye	Q_max_ (mg/g)	K (L/mol)	*R* ^2^	N
OSS	898.4 ± 15.6	0.06059 ± 0.00500	0.978	33
NBB	240.2 ± 10.0	0.35811 ± 0.07050	0.930	24
BB	228.7 ± 6.9	0.13381 ± 0.02544	0.875	39

**Table 4:** Results of *pseudo second-order* fitting on all kinetic datasets. The number of experimental data used for each dye is reported in column N.

Pseudo Second-Order Model					
Dye	C_0_	k_2_	q_eq_	*R* ^2^	N
		mg·g^−1^·min^−1^	mg·g^−1^		
OSS	20 mg/L	8.9 × 10^−3^	25	0.9999	30
	800 mg/L	5.7 × 10^−4^	500	0.9998	30
NBB	20 mg/L	1.3 × 10^−2^	20	0.9997	30
	250 mg/L	2.6 × 10^−5^	200	0.9887	30
BB	100 mg/L	4.0 × 10^−4^	100	0.9989	24
	320 mg/L	1.9 × 10^−4^	167	0.9989	24

**Table 5:** Sorption–desorption cycles: sorption capacity and equilibrium concentration after each regenerating cycle, considering OSS dye (C_0_ = 20 mg/L).

Iterations	1	2	3	4	5
C_e_ (mg/L)	3.13 ± 0.22	2.59 ± 0.19	1.65 ± 0.16	2.06 ± 0.41	3.21 ± 0.35
q_e_ (mg/g)	21.78 ± 1.19	22.17 ± 1.06	24.41 ± 0.98	24.54 ± 1.61	21.84 ± 1.02

**Table 6:** Comparison of sorption capacities (q_e_, (mg/g)) determined at C_0_ low and C_0_ high for SAE SUPER and CNS (for C_0_ values see Table 1).

Sorbent	OSS		NBB		BB		CBY	
	C_0_ Low	C_0_ High	C_0_ Low	C_0_ High	C_0_ Low	C_0_ High	C_0_ Low	C_0_ High
SAE SUPER	25.6 ± 2.0	383.7 ± 3.6	23.8 ± 1.9	220.1 ± 2.8	108.7 ± 2.2	360.3 ± 3.6	118.0 ± 2.1	159.8 ± 3.6
CNS	22.6 ± 1.6	793.4 ± 5.1	23.9 ± 1.8	239.0 ± 3.2	89.0 ± 1.9	197.0 ± 3.8	121.5 ± 2.3	242.1 ± 2.9

## Supplementary Materials

The following are available online at https://www.mdpi.com/2079-4991/10/8/1570/s1. Detailed TOCNF preparation; Detailed synthesis of CNS; Procedure for determination of calibration lines; Isotherm models (Langmuir model, Freundlich model, Dubinin-Radushkevich model); *Pseudo second-order* fitting; General characteristics of NORIT^®^ SAE SUPER. Figure S1: UV-vis spectra for (A) OSS, (B) NBB, (C) BB and (D) CBY; Figure S2: Calibration line for Orange Sodium Salt; Figure S3: Calibration line for Naphtol Blue Black; Figure S4: Calibration line for Brilliant Blue R; Figure S5: Calibration line for Cibacron Brilliant Yellow; Figure S6: Isotherm fitting with Langmuir model; Figure S7: Isotherm fitting with Freundlich model; Figure S8: Isotherm fitting with Dubinin-Radushkevic model. Figure S9: OSS kinetic at 20 mg/L; Figure S10: OSS kinetic at 800 mg/L.Table S1: Characteristic UV-vis peaks of each dye; Table S2: Molar mass and extinction coefficient of each dye; Table S3: Values of initial concentrations (C_0_) for isotherm curves expressed as mg/L; Table S4: Estimation of Qmax and K parameters according to the Langmuir isotherm model for OSS, NBB and BB; Table S5: Estimation of Qmax and K parameters according to the Freundlich isotherm model for OSS, NBB and BB; Table S6: Estimation of Qmax and K parameters according to the Dubinin-Radushkevic isotherm model for OSS, NBB and BB; Table S7: Summary of k and q_eq_ values obtained with *pseudo first-order* and *pseudo second-order* fitting of the kinetic data set.
