# Supplementary material for: Nanostructured Cellulose-Based Sorbent Materials for Water Decontamination from Organic Dyes
Source: Nanomaterials (Basel). 2020 Aug 10;10(8):1570. doi: 10.3390/nano10081570 (PMC7466597; doi:10.3390/nano10081570)
Supplement: Supplementary file 1 [file nanomaterials-10-01570-s001.pdf]

nanomaterials

MDPI

Article

Nanostructured Cellulose-Based Sorbent Materials for Water Decontamination from Organic Dyes

Laura Riva [1], Nadia Pastori [1], Alice Panozzo [1], Manuela Antonelli [2,*] and Carlo Punta [1,3,*]

[1] Department of Chemistry, Materials, and Chemical Engineering "G. Natta", Politecnico di Milano, Piazza Leonardo da Vinci 32, I-20133 Milano, Italy, and INSTM Local Unit; laura2.riva@polimi.it (L.R.); nadia.pastori@polimi.it (N.P.); alice.panozzo@mail.polimi.it (A.P.)

[2] Department of Civil and Environmental Engineering, Politecnico di Milano, Piazza Leonardo da Vinci 32, I-20133 Milano, Italy

[3] Centro Nazionale Ricerche (C. N. R.) Istituto di Chimica del Riconoscimento Molecolare (ICRM), Milan, Italy

[*] Correspondence: manuela.antonelli@polimi.it (M.A.); carlo.punta@polimi.it (C.P.); Tel: 0039-0223-996-407 (M.A.); 0039-0223-993-026 (C.P.).

SUPPLEMENTARY MATERIALS

SUMMARY

1. **Detailed TOCNF Preparation……………………..……………………………………………S1**
2. **Detailed synthesis of CNS……………………..…………………………...……….…S2**
3. **Calibration lines…………………………………………………………………………...S3**
4. **Isotherms………………...……………………….……………..……………………………… S6**
5. **Isotherm models……………………….....…………………………………………….……….. S6**
6. **Kinetic models...…...……………..……………………….…………………...…………….. S8**
7. ***Pseudo second-order* fitting………………………………………………………………......S8**
8. **General Characteristics of NORIT® SAE SUPER……………………………...…..…S10**

1. Detailed TOCNF Preparation

Synthesis

A total of 100 g of cotton linter-derived cellulose was ground with the aid of a domestic mixer in a 2 L beaker; deionized water was added to facilitate the grinding procedure. Meanwhile, 2.15 g of TEMPO ((2,2,6,6-Tetramethylpiperidin-1-yl)oxyl) and 15.42 g of KBr were dissolved in 2 L of deionized water under stirring in an 8 L keg. The water-dispersed paper, once homogenized, was transferred in the keg and water was added until the total volume of 5.7 L was reached.

While keeping the solution stirred, a pH meter and two dropping funnels were installed above the keg. One of the funnels contained 437 mL of 12% NaClO aqueous solution. and the other 250 mL of NaOH 4 M. NaClO was then dripped into the solution while monitoring the pH to maintain it above 10.5-11 by dripping 4 N NaOH. This is the pH range at which the TEMPO-oxidation reaction is favored. Once the NaClO dripping was completed, pH was still maintained in the same range by dripping 4 N NaOH until stability was reached. After that, the solution was stirred for 16 hours. Following this, the oxidized cellulose was acidified by using 37% w/v HCl with the purpose of aggregating the cellulose fibers to simplify their separation from water by filtration. The slurry obtained was filtered by means of a Büchner funnel and washed with deionized water (2 L, 5-6 times). Final washes were performed with acetone (0.5 L, 2 times) in order to remove the water and

allow a rapid drying of the cellulose. Cellulose was then dried in natural air. The measured weight of dried oxidized cellulose was about 83.8 g, achieving an almost 84% yield on the initial paper cellulose weight.

Titration

To estimate the concentration of carboxyl groups on the cellulose structure after oxidation, titration was performed with NaOH using phenolphthalein as colorimetric indicator. To begin with, an NaOH solution was titrated by means of potassium hydrogen phthalate. A solution was prepared by dissolving 218.6 mg (1.0704 mmol) of phthalate in water and by adding 2-3 drops of phenolphthalein solution 10 mM in acetonitrile. Then, the NaOH solution was dripped in the beaker under continuous stirring. Neutralization of the phthalate occurred when 11.35 mL of NaOH was dripped in the solution. The molar concentration of the solution was calculated to be 0.0943 M. This solution was then used to titrate the oxidized cellulose obtained with the TEMPO/NaClO/KBr system. A TOCNF water dispersion was prepared by adding 616.0 mg of TOCNF to deionized water and sonicating it to improve the grade of dispersion. A drop of the same phenolphthalein solution described in the previous paragraph was added. The NaOH solution, previously titrated, was then dripped in the beaker under continuous stirring: 10.21 mL was required to neutralize the carboxyl acids of the oxidized cellulose. The concentration of the carboxyl groups was calculated by means of the following equation

$$[COOH] = \frac{M_{NaOH,sol} * x_{COOH} * V_{NaOH,sol}}{m_{TOCNF}} \quad (1)$$

where $M_{NaOH,sol}$ is the moles of NaOH in 1 L of solution, x_{COOH} is the molar fraction of COOH per mol of NaOH, $V_{NaOH,sol}$ is the dripped volume of NaOH solution and m_{TOCNF} is the titrated mass of TOCNF.

2. Detailed synthesis of CNS

In a 1 L beaker, 3.5 g of oxidized cellulose was suspended in 140 mL deionized water, in order to obtain a 2.5% w/v solution. While mixing it, granular NaOH in a stoichiometric quantity was added to the solution to obtain basic pH (evaluated with litmus paper), obtaining a slightly yellow viscous solution. The beaker was placed in an ice bath and the solution was sonicated (working at 20 kHz in continuous mode with an output power 50% the nominal value (200 W)) to further promote the separation of the nanofibers, obtaining a transparent and more homogeneous solution. The latter was then acidified with concentrated HCl and filtered on a Büchner funnel under vacuum, then washed and re-suspended. This procedure was repeated with deionized water until a neutral pH was reached. Wet cellulose was removed from the filter paper and weighted; 3.5 g was assumed to correspond to the starting cellulose, while the remaining mass represented the residual water content. The remaining water necessary to obtain a 2.8-3% w/v TOCNFs solution for the next phase was split up in three quotas, in order to re-suspend the cellulose and dissolve the reticulant polymer (branched polyethylenimine, bPEI 25kDa) and the co-reticulant agent (citric acid monohydrate), in three separate batches. The amount of the co-reticulant was calculated by means of the following equation

$$m_{CA} = x\, g_{bPEI} * 7.4 \frac{mmol_{NH_2}}{g_{bPEI}} * 0.18 \frac{mmol_{CA}}{mmol_{NH_2}} * \frac{mmol_{CA*1H_2O}}{mmol_{CA}} * 192.12 \frac{mg_{CA}}{mmol_{CA}} = y\, mg_{CA} \quad (2)$$

Once dissolved in water, the reticulant and the co-reticulant agents were slowly added to the cellulose solution, while continuously stirring until obtaining a white and homogeneous hydrogel, which was placed in 24-well well-plates (about 15.6 mm diameter, half-filled with about 2 mL each) and quickly frozen at -35°C. After a few hours, the well-plates were moved to the lyophilizer, which allowed water removal by sublimation, thus retaining the internal structure of the frozen hydrogels. At the end of the process, white cylindrical-shaped spongy aerogels were obtained. They were removed from the wells and placed in the laboratory oven, at the initial temperature of 55°C. The temperature was then slowly raised up to 102°C in 4 hours, in order to avoid sudden overheating and prevent the risk of burning the material. Above 100°C, the water formed due to the reticulation reactions between carboxylic moieties and ammines (creation of amidic bonds) was quickly evaporated, allowing the proper reticulation of the material. The sponges were removed

from the oven after 16 hours. Their aspect was yellow and their texture slightly soft, but it hardened quickly while cooling down. If not kept in an anhydrous atmosphere, they tended to sorb water and become softer. At the end of the process, CNSs were washed six times with deionized water and the last time with ethanol to eliminate the excess of bPEI.

3. Calibration lines

To evaluate the sorption capacity of the sponges, the absorbance of a water solution of the dyes was measured before and after every sorption cycle to calculate the amount of dye sorbed by the sponge during each cycle. To build a quantitative correlation between absorbance readings and dye concentration, a calibration line was built for each dye. First, a spectrum analysis for each dye was carried out to identify their characteristic peaks. The resulting selected peaks are summarized in **Table S1**. Spectra for each dye are reported in **Figure S1**.

Table S1. Characteristic UV-vis peaks of each dye.

Dye	OSS	NBB	BB	CBY
**λ [nm]**	485	618	588	402

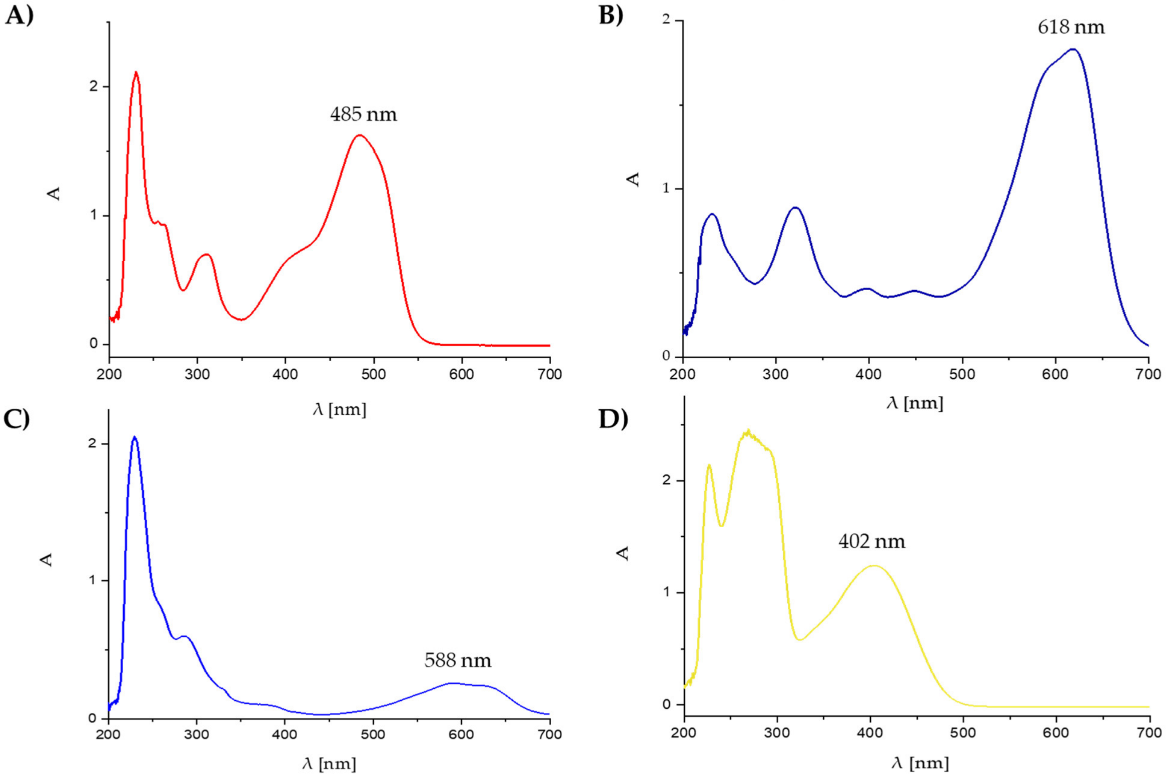

Figure S1. UV-vis spectra for **A)** OSS, **B)** NBB, **C)** BB and **D)** CBY.

A total of 1 L of 100 mg/L solution was prepared. At least five solutions with different known concentrations were obtained for each dye by preparing various dilutions of the first solution. Single-wavelength quantitative absorbance readings were performed for each solution. Absorbance was plotted against concentration to find the interval of applicability of the Lambert–Beer law. The straight line passing through the measured points in the A vs. C graph was plotted; the slope value was then divided by the molar mass of the dye molecule to obtain the extinction coefficients, which are reported in **Table S2**. Calibration lines Absorbance vs. Concentration (mg/L) for all the considered dyes are reported in **Figure S2, S3, S4** and **S5.**

Table S2. Molar mass and extinction coefficient of each dye.

Dye	OSS	NBB	BB	CBY
**Molar Mass [g/mol]**	350.32	616.50	825.97	624.55
ε	21.998	49.377	7.828	7.537

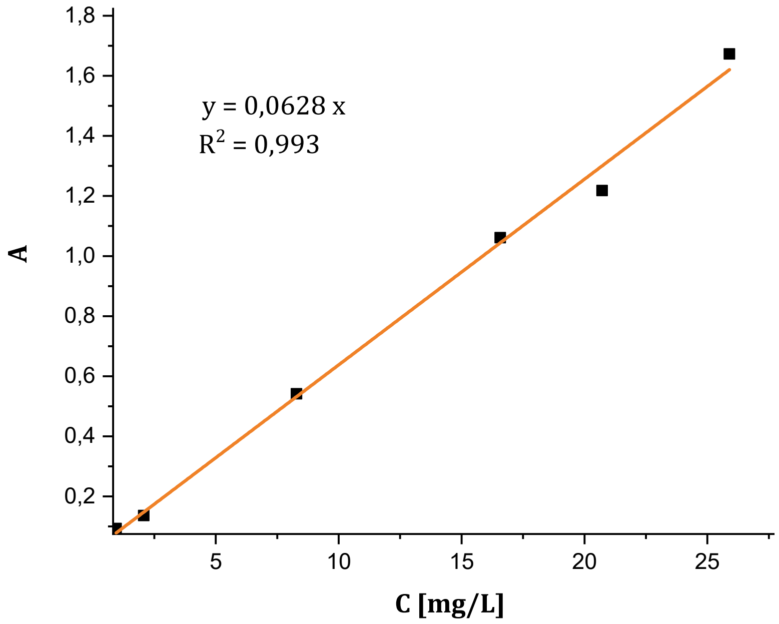

Figure S2. Calibration line for Orange Sodium Salt.

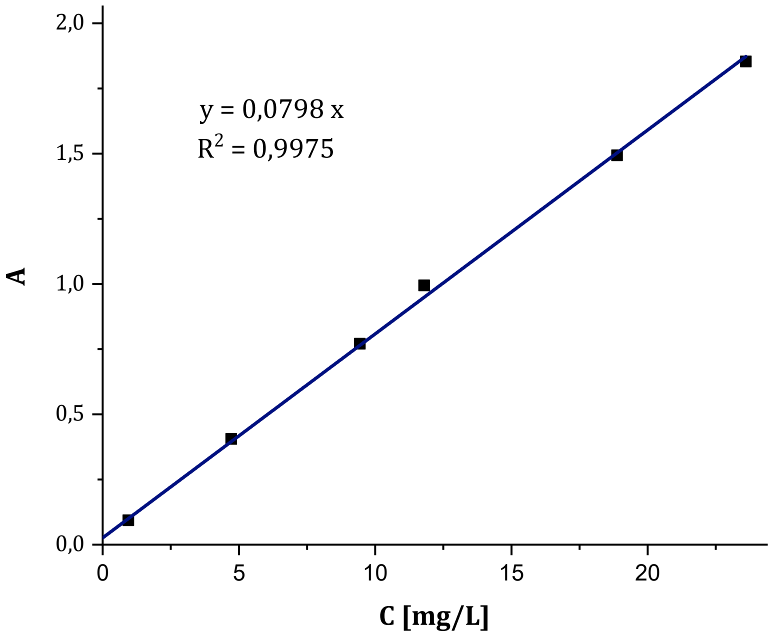

Figure S3. Calibration line for Naphtol Blue Black.

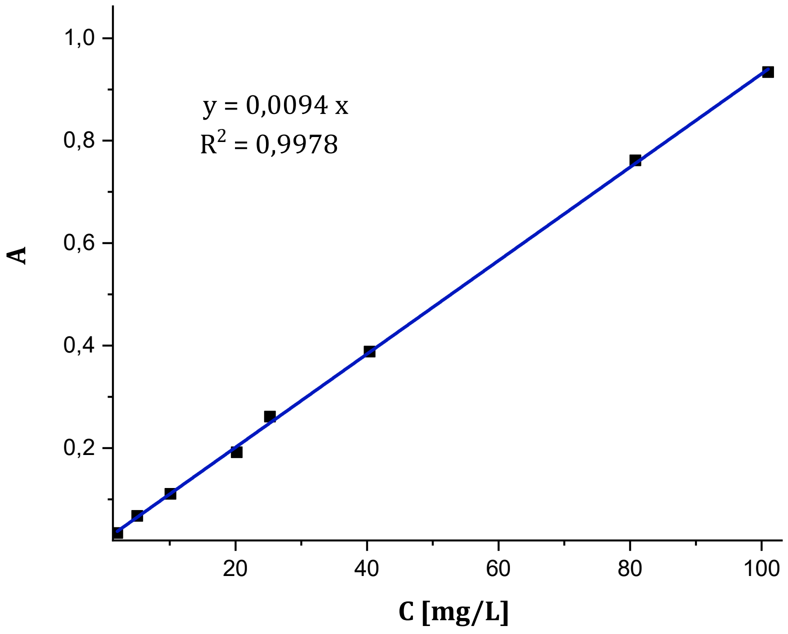

Figure S4. Calibration line for Brilliant Blue R.

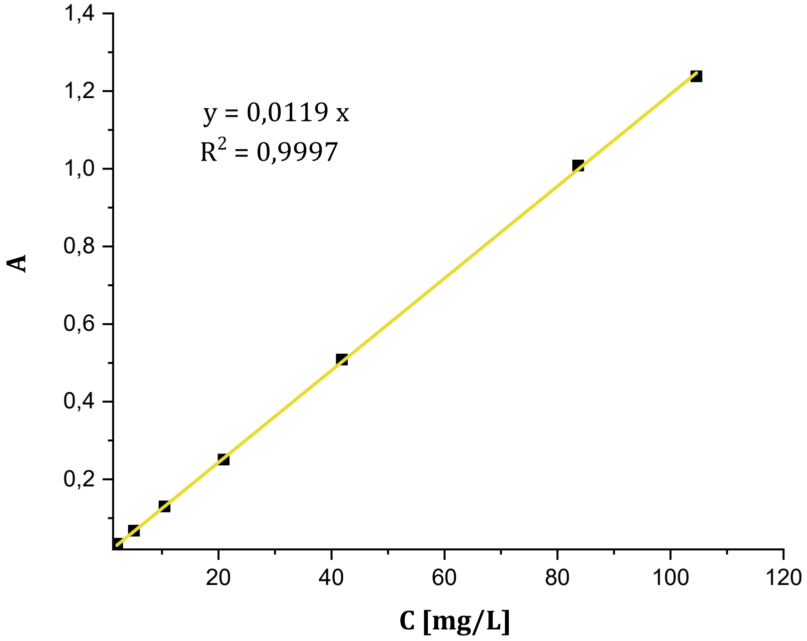

Figure S5. Calibration line for Cibacron Brilliant Yellow.

4. Isotherms

nanomaterials

MDPI

Table S3. Values of initial concentrations (C_0) for isotherm curves expressed as mg/L.

	Initial concentration C_0 [mg/L]																
**OSS**	**10**	**-**	**50**	**100**	**-**	**200**	**-**	**260**	**-**	**320**	**480**	**640**	**700**	**800**	**920**	1000	1200
**NBB**	10	20	50	100	150	200	230	260	-	320	480	-	-	-	-	-	-
**BB**	-	20	50	100	150	200	-	260	-	320	480	640	-	800	-	1000	-
**CBY**	10	-	50	100	-	200	-	260	280	320	480	-	700	800	-	-	-

5. Isotherm models

Langmuir model

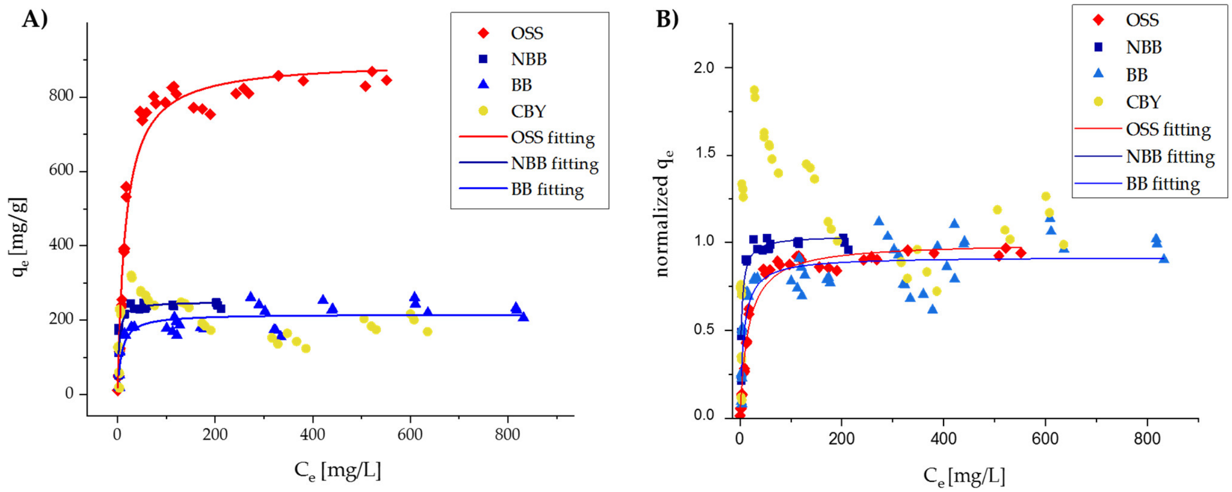

Figure S6. Isotherm fitting with Langmuir model. The x-axis shows the concentration at equilibrium (C_e) expressed in mg/L, while the y-axis shows the capacity at equilibrium (q_e) expressed in mg/g and not normalized (A) and normalized for the maximum capacity (Q_{max}) (**B**).

Table S4. Estimation of Qmax and K parameters according to the Langmuir isotherm model for OSS, NBB and BB.

Dye	Q_{max} [mg/g]	K [L/mol]	R^2	*N*
**OSS**	898.4 ± 15.6	0.06059 ± 0.00500	0.978	33
**NBB**	240.2 ± 10.0	0.35811 ± 0.07050	0.930	24
**BB**	228.7 ± 6.9	0.13381 ± 0.02544	0.875	39

Freundlich model

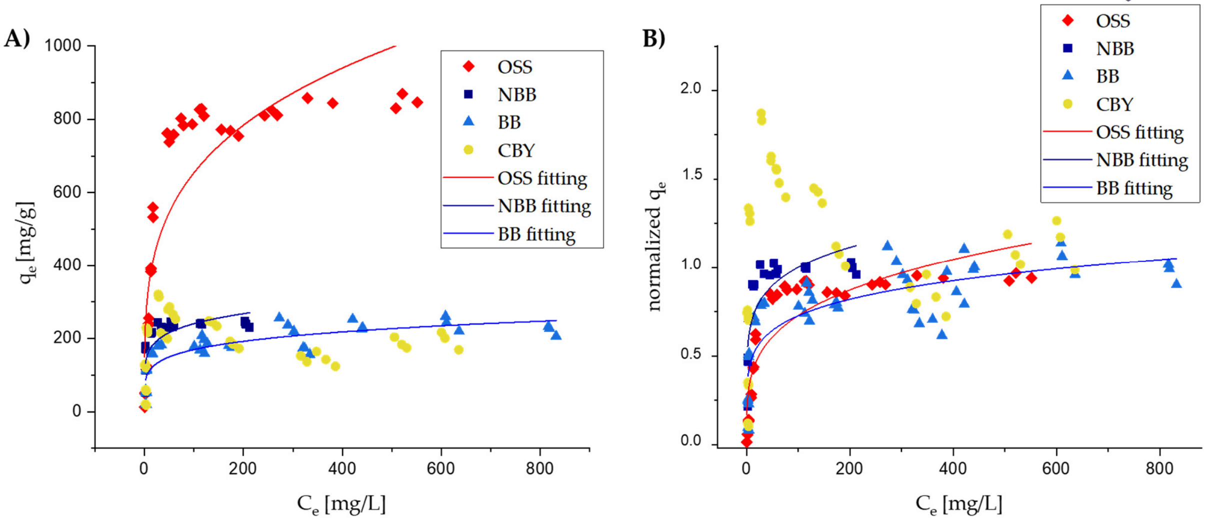

Figure S7. Isotherm fitting with Freundlich model. The x-axis shows the concentration at equilibrium (C_e) expressed in mg/L, while the y-axis shows the capacity at equilibrium (q_e) expressed in mg/g and not normalized (A) and normalized for the maximum capacity (Q_{max}) (**B**).

Table S5. Estimation of Qmax and K parameters according to the Freundlich isotherm model for OSS, NBB and BB.

Dye	K_f [(L/mol)$^{1/n}$·g^{-1}]	n	R^2	*N*
**OSS**	195.9 ± 27.5	3.82025 ± 0.39812	0.821	33
**NBB**	117.2 ± 13.3	6.41263 ± 1.17012	0.708	24
**BB**	73.5 ± 9.3	5.51221 ± 0.69909	0.712	39

Dubinin-Radushkevic model

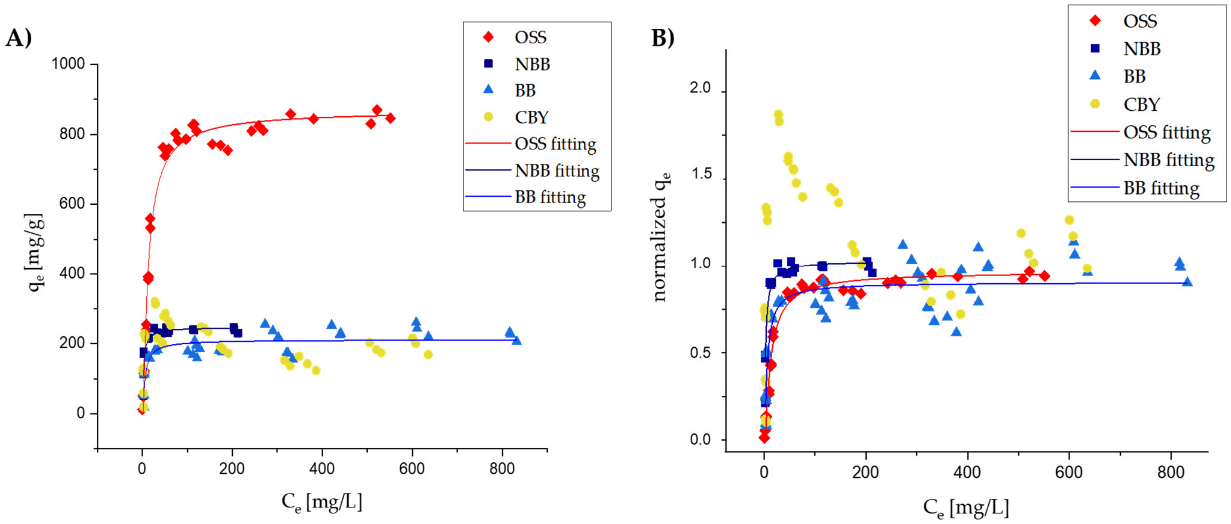

Figure S8. Isotherm fitting with Dubinin-Radushkevic model. The x-axis shows the concentration at equilibrium (C_e) expressed in mg/L, while the y-axis shows the capacity at equilibrium (q_e) expressed in mg/g and not normalized (A) and normalized for the maximum capacity (Q_{max}) (**B**).

Table S6. Estimation of Qmax and K parameters according to the Dubinin-Radushkevic isotherm model for OSS, NBB and BB.

Dye	q_s [mol/g]	K [mol²/J²]	R^2	*N*
**OSS**	869.5 ± 9.3	10.07999 ± 0.40697	0.988	33
**NBB**	247.4 ± 9.5	2.22612 ± 0.30369	0.832	24
**BB**	211.7 ± 6.3	5.00380 ± 0.66962	0.794	39

6. Kinetic models

Table S7. Summary of k and q_{eq} values obtained with pseudo first-order and pseudo second-order fitting of the kinetic data set.

		Pseudo first-order			*Pseudo second-order*		
		k_1	q_{eq}	R^2	k_2	q_{eq}	R^2
		**min⁻¹**	mg·g⁻¹		mg·g⁻¹·min⁻¹	mg·g⁻¹	
**OSS**	**20 mg/L**	**$3.45 \cdot 10^{-2}$**	12	0.9022 [a]	$8.9 \cdot 10^{-3}$	25	0.9999 [c]
	800 mg/L	$4.00 \cdot 10^{-3}$	339	0.9321 [a]	$5.7 \cdot 10^{-4}$	500	0.9998 [c]
**NBB**	20 mg/L	-	-	-	$1.3 \cdot 10^{-2}$	20	0.9997 [c]
	250 mg/L	$4.00 \cdot 10^{-3}$	126	0.9989 [a]	$2.6 \cdot 10^{-5}$	200	0.9887 [c]
**BB**	100 mg/L	$1.15 \cdot 10^{-2}$	63	0.9958 [b]	$4.0 \cdot 10^{-4}$	100	0.9989 [c]
	320 mg/L	$8.75 \cdot 10^{-3}$	75	0.9827 [b]	$1.9 \cdot 10^{-4}$	167	0.9989 [c]

[a] Considering only the first part of the dataset.
[b] Considering only the central part of the dataset.
[c] Considering the whole dataset.

7. Pseudo second-order fitting

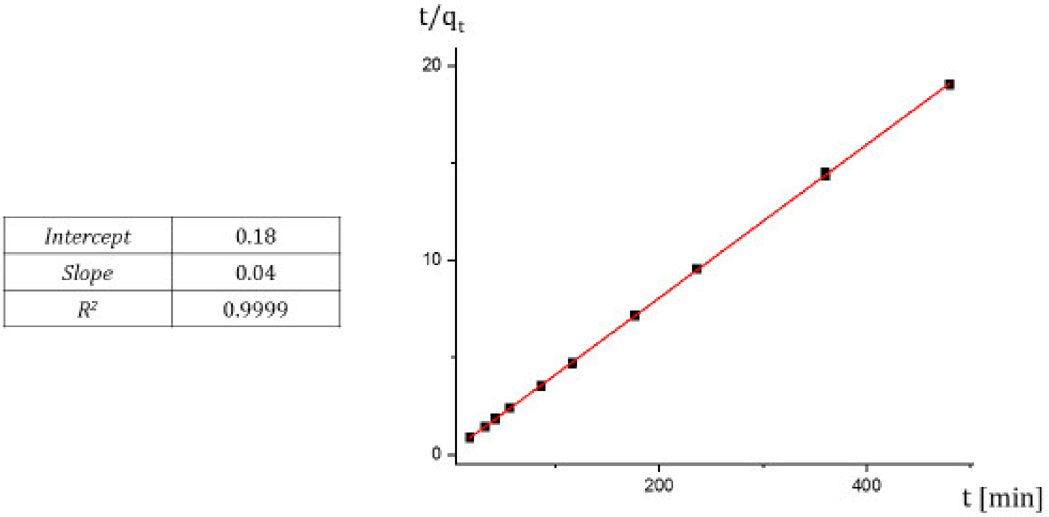

Figure S9. OSS kinetic at 20 mg/L

Equation:

Equation:

$$\frac{dq_t}{dt} = k_2\left(q_{eq} - q_t\right)^2 \tag{3}$$

Linearized equation:

$$\frac{t}{q_t} = \frac{1}{k_2 q_{eq}^2} + \frac{1}{q_{eq}} t \quad (4)$$

$x = t$

$y = \frac{t}{q_t}$

$m = \frac{1}{q_{eq}} = 0.04$

$q = \frac{1}{k_2 q_{eq}^2} = 0.18$

$q_{eq} = 25$ mg·g^{-1}

$k_2 = 0.0089$ mg·g^{-1}·min^{-1}

$R^2 = 0.9999$

Intercept	0.007
Slope	0.002
R²	0.9998

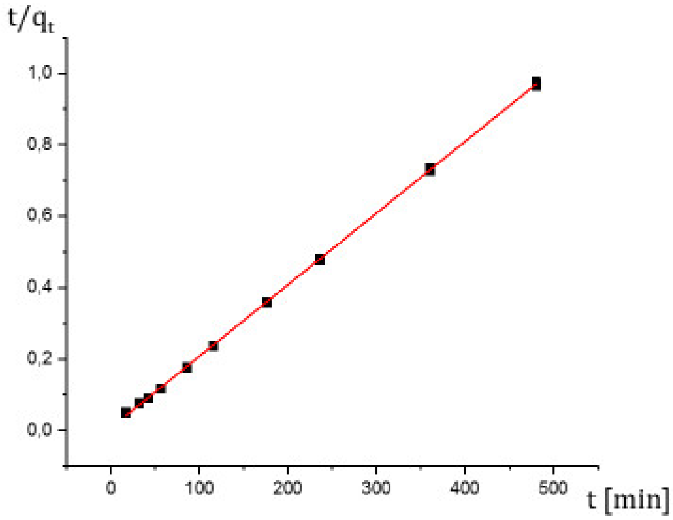

Figure S10. OSS kinetic at 800 mg/L

Equation:

$$\frac{dq_t}{dt} = k_2 (q_{eq} - q_t)^2 \quad (5)$$

Linearized equation:

$$\frac{t}{q_t} = \frac{1}{k_2 q_{eq}^2} + \frac{1}{q_{eq}} t \quad (6)$$

$x = t$

$y = \frac{t}{q_t}$

$$m = \frac{1}{q_{eq}} = 0.002 \qquad q = \frac{1}{k_2 q_{eq}^2} = 0.007$$

$q_{eq} = 500$ mg·g^{-1}

$k_2 = 0.00057$ mg·g^{-1}·min^{-1}

$R^2 = 0.9998$

8. General Characteristics of NORIT® SAE SUPER

Iodine number	*min. 950*
Total surface area (B.E.T.)	*1050 m^2/g*
Apparent density, tamped	*375 kg/m^3*
Particle size > 150 µm	*3 mass-%*
Particle size, D_{50}	*20 µm*
